# Editorial: Cancer Immunosurveillance

**DOI:** 10.3389/fimmu.2026.1897386

**Published:** 2026-06-12

**Authors:** Sam Hanash, Luigi Buonaguro, Olivera Finn

**Affiliations:** 1 Department of Clinical Cancer Prevention, MD Anderson Cancer Center, Houston, TX, United States; 2 Lab of Innovative Immunological Models, National Cancer Institute - IRCCS ‘Pascale’, Naples, Italy; 3 Department of Immunology, University of Pittsburgh, Pittsburgh, PA, United States

**Keywords:** cancer, immunosurveillance, prevention, tumor antigens, vaccine

Elucidation of cancer immunosurveillance processes has provided a scientific framework for how immune systems eliminate tumors, keep them in check, or fail resulting in immune evasion. This understanding has provided a foundation for immunotherapy as well as for cancer risk assessment, preventive intervention and prognostication. Some of the resulting innovations are exemplified in reviews and in original articles submitted to this Research Topic on Cancer Immunosurveillance.

A study by Jin et al. elucidated that T cell-mediated immune surveillance conferred by latent Epstein-Barr virus genes suppressed a broad spectrum of tumor formation through NKG2D-NKG2DL interactions. In this context despite nearly absent MHC class I expression, tumor cells were effectively eliminated by CD8 + LMP1/2A induced T cells that recognized commonly expressed tumor associated antigens (TAA) in T-cell malignancies and in intestinal tumors caused by Apc Min mutation. The findings implicate a beneficial result of EBV infection in humans and point to some cancer protective effect of exposure to infectious diseases. This concept is also supported in another study that investigated molecular mimicry of SARS-COV-2 antigens as a possible natural anti-cancer preventive immunization by Ragone et al. Significant homologies in amino acidic sequence have been found between SARS-CoV-2 peptides and multiple TAAs in breast, liver, melanoma and colon cancers. The study provides evidence that several SARS-COV-2 antigens are highly homologous to TAAs and cross-reactive T cells are identified in infected and BNT162b2 preventively vaccinated individuals. Follow up studies of infected or vaccinated individuals are needed to confirm anti-cancer benefit.

A provocative article by Nemchinov describes a mechanism in plants for defense against pathogens called non-host resistance (NHR), through which a species is resistant to all isolates of a microbial species. The author’s hypothesis based on current knowledge, is that it would be possible to render an animal organism an absolute non-host for cancer through a cancer hostile environment.

In another study by Ewaisha et al., serum profiling of the antibody response to HPV was assessed in women with or without abnormal cervical cytology undergoing cervical cancer screening. The findings highlighted the kinetics of HPV-specific humoral immunity in women with normal or abnormal cervical cytology and emphasized the need for comprehensive immune profiling at various disease stages.

Mining humoral immune response to tumor antigens has potential value for diagnostic applications. A study by Mowoe et al. identified autoantibodies to 11 antigens with specificity to pancreatic ductal adenocarcinoma. The clinical utility of the biomarkers was tested in an independent cohort which resulted in a panel consisting of CEACAM1-DPPA2-DPPA3-MAGEA4-SRC-TPBG-XAGE3 as a promising combination for clinical application.

On the immune evasion side, a review by He et al. addresses the mechanisms through which chronic stress, promotes tumor progression, disrupting anti-tumor immunity. Stress hormones alter the tumor microenvironment and impair immune surveillance through suppressed activity of cytotoxic lymphocytes and expansion of immunosuppressive cells leading to immune evasion. The review highlights the promise of targeting stress signaling, through both pharmacological and behavioral interventions.

On the prevention side, there is much interest in the use of vaccines to strengthen cancer immunosurvaillance in order to reduce the incidence of cancer among individual at risk either because of inherited susceptibility or because of other risk factors. There is a multitude of ways to design and deliver a vaccine for individuals at risk. An example of vaccine design is presented in the article by Bansal et al. Lynch syndrome (LS) is a hereditary cancer syndrome associated with increased risk for colorectal and other cancers. A vaccine was designed against tumor-associated antigens CEA, MUC1, and brachyury, delivered in an adenovirus vector combined with an immune-enhancing IL-15 receptor super agonist. A study of pre-vaccination transcriptomic profiles of immune responders to a MUC1 peptide vaccine for colon cancer prevention by Cameron et al. found that MUC1 vaccine responders had higher frequencies of CD4 cells pre-vaccination, increased expression of CD40L on CD8 and CD4 T-cells, and a greater increase in ICOS expression on CD8 T-cells. Differential gene expression analysis revealed that iCOSL, PI3K AKT MTOR, and B-cell signaling pathways are activated early in response to the MUC1 vaccine. These findings suggested that some individuals may be predicted to respond better than others based on their peripheral blood immune cell profile.

Another article by Johnson and Disis in this Research Topic reviewed the current state of vaccines to prevent cancer in individuals with familial adenomatous polyposis (FAP) an inherited disorder associated with the development of adenomas in the small and large intestines. The review highlights the challenges and opportunities of intercepting cancer in FAP by means of vaccines.

This timely Research Topic highlights only some of ever increasing contributions to our understanding of immunosurveillance that results in cancer interception and prevention. The findings presented in the research articles and reviews highlight the progress made to date with a potential for clinical applications. In particular, the interconnection between infections and cancer as well as the role of pre-existing immunity to tumor antigens shed lights on relevant mechanisms of immunosurveillance.

